# Endothelial activation and stress index (EASIX) is a reliable predictor for overall survival in patients with multiple myeloma

**DOI:** 10.1186/s12885-020-07317-y

**Published:** 2020-08-24

**Authors:** Ga-Young Song, Sung-Hoon Jung, Kihyun Kim, Seok Jin Kim, Sang Eun Yoon, Ho Sup Lee, Mihee Kim, Seo-Yeon Ahn, Jae-Sook Ahn, Deok-Hwan Yang, Hyeoung-Joon Kim, Je-Jung Lee

**Affiliations:** 1 Department of Hematology-Oncology, Chonnam National University Hwasun Hospital, 322 Seoyangro, Hwasun, Jeollanamdo 519-763 Republic of Korea; 2 Division of Hematology-Oncology, Department of Medicine, Samsung Medical Center, Sungkyunkwan University School of Medicine, 81 Irwon-ro, Gangnam-gu, Seoul, Republic of Korea; 3 Kosin University Gospel Hospital, Busan, Republic of Korea

**Keywords:** Multiple myeloma, Prognosis, LDH, Platelets, Serum creatinine

## Abstract

**Background:**

Recently, the endothelial Activation and Stress Index (EASIX) score has been reported to predict overall survival (OS) after allogeneic stem cell transplantation. This study evaluated the prognostic role of EASIX score in patients with newly diagnosed multiple myeloma (MM).

**Methods:**

This retrospective study analyzed the records of 1177 patients with newly diagnosed MM between February 2003 and December 2017 from three institutions in the Republic of Korea. Serum lactate dehydrogenase (LDH), creatinine, and platelet count at diagnosis were measured in all included patients. EASIX scores were calculated using the formula-LDH (U/L) × Creatinine (mg/dL) / platelet count (10^9^/L) and were evaluated based on log2 transformed values.

**Results:**

The median age of patients was 63 years (range, 22–92), and 495 patients (42.1%) underwent autologous stem cell transplantation (ASCT). The median log2 EASIX score at diagnosis was 1.1 (IQR 0.3–2.3). Using maximally selected log-rank statistics, the optimal EASIX cutoff value for OS was 1.87 on the log2 scale (95% CI 0.562–0.619, *p* < 0.001). After median follow-up for 50.0 months (range, 0.3–184.1), the median OS was 58.2 months (95% CI 53.644–62.674). Overall, 372 patients (31.6%) showed high EASIX scores at diagnosis, and had significantly inferior OS compared to those with low EASIX (log2 EASIX ≤1.87) (39.1 months vs. 67.2 months, *p* < 0.001). In multivariate Cox analysis, high EASIX was significantly associated with poor OS (HR 1.444, 95% CI 1.170–1.780, *p* = 0.001). In the subgroup analysis of patients who underwent ASCT, patients with high EASIX showed significantly inferior OS compared to those with low EASIX (52.8 months vs. 87.0 months, *p* < 0.001). In addition, in each group of ISS I, II, and III, high EASIX was associated with significantly inferior OS (ISS 1, 45.2 months vs. 76.0 months, *p* = 0.001; ISS 2, 42.3 months vs. 66.5 months, *p* = 0.002; ISS 3, 36.8 months vs. 55.1 months, *p* = 0.001).

**Conclusion:**

EASIX score at diagnosis is a simple and strong predictor for OS in patients with newly diagnosed MM.

## Background

Multiple myeloma (MM) is a monoclonal plasma cell proliferation disorder with various symptoms and signs caused by monoclonal proteins [1]. Recent molecular studies have shown that MM is a genetically heterogeneous disease. In addition, clonal evolution and additional genetic events during the disease course affect the progression of the asymptomatic state to symptomatic disease and lead to a refractory disease state [2, 3]. Therefore, MM remains an incurable disease and shows various survival outcomes despite the development of new effective agents such as immunomodulatory drugs (IMids), proteasome inhibitors, and monoclonal antibodies. The most common staging system to predict the prognosis of MM is the Revised International Staging System (R-ISS) [4]. The R-ISS was created by incorporating the chromosomal abnormalities and serum lactate dehydrogenase (LDH) into the original ISS and improved the prognostic power compared with the original ISS, cytogenetics, and LDH alone. However, cytogenetic abnormalities in the R-ISS do not include all the genetic abnormalities in MM and can be only assessed in selected institutions that can conduct gene analysis. For these reasons, there are still unmet needs about establishing a precise and convenient risk stratification model for MM.

Recently, a Germany and the United States (US) group presented the Endothelial Activation and Stress Index (EASIX), which is calculated by the formula-LDH (U/L) × Creatinine (mg/dL) / platelet count (10^9^/L) as a reliable factor to predict the prognosis of acute graft-versus-host disease after allogeneic stem cell transplantation [5]. They subsequently proposed that EASIX could predict the survival outcome in lower-risk myelodysplastic syndrome which is not a candidate for allogeneic stem cell transplantation [6]. The prognostic impact of EASIX in allogeneic stem cell transplantation was externally validated in generalized population cohorts [7–9]. Platelet count, serum creatinine and LDH, which make up EASIX, are well-known prognostic factors for MM. Therefore, we planned this study to determine whether EASIX could also be useful to predict the survival outcomes for MM.

## Methods

### Patients

For this retrospective study, we analyzed the records of 1260 patients with newly diagnosed MM between February 2003 and December 2017 from three institutions in the Republic of Korea. Monoclonal gammopathy of undetermined significance (MGUS), non-secretory MM, amyloidosis, and plasma cell leukemia were excluded. Additionally, 83 patients with lack of laboratory data such as serum LDH, serum creatinine or platelet count at diagnosis were excluded and finally 1177 patients were included in the analysis. The study was approved by the Institutional Review Board of each participating institution and was conducted in accordance with the Declaration of Helsinki.

### ISS and EASIX analysis

ISS, R-ISS, and EASIX were assessed at initial diagnosis. Chromosomal abnormalities (CA) were evaluated based on conventional cytogenetic studies or fluorescence in situ hybridization. High-risk CA was characterized by the presence of at least one of del(17p), t(4;14), or t(14;16). Standard-risk CA was characterized by the absence of previously mentioned abnormalities. EASIX score was calculated by the formula-LDH (U/L) × Creatinine (mg/dL) / platelet count (10^9^/L) and evaluated based on log2 transformed values.

### Statistical analysis

Pearson’s χ^2^ test and the Mann–Whitney *U* test were used for discrete and continuous variables to compare the patient characteristics. The primary end point was overall survival (OS), defined as the time from diagnosis to death as a result of any cause, or to the last follow-up date. The Kaplan-Meier method was used to estimate the OS, and the survival curves were compared using a log-rank test. Maximally selected log-rank statistics using exactGauss [10] were applied to calculate an optimal cutoff in survival distributions according to EASIX. The prediction error curves and concordance index curves are estimated using the statistical software R (version 3.3.3), together with R packages survival (version 2.41–2), prodlim (version 1.6.1), maxstat (version 0.7–25), riskRegression (version 1.1.7) and pec (version 2.5.3) for statistical calculation. The estimate of the relative risk of an event and its 95% confidence interval (95% CI) for OS were assessed by univariate and multivariate analyses using a Cox proportional hazard model. The Cox proportional hazard model was calculated using log2 transformed index of EASIX It means that a hazard ratio of 1.25 corresponds to a 25% increase of the hazard for a two-fold increase of EASIX or one-fold increase of log2(EASIX). All statistical computations were performed using SPSS software (ver. 25; SPSS Inc., Chicago, IL, ISA) and R (version 3.3.3). A *p*-value < 0.05 was considered significant in all of the analyses.

## Results

### Patient characteristics and treatments

The median age of the patients was 63.0 years (range, 22.0–92.0), and 44.9% were older than 65 years. The most prevalent MM type was IgG (54.9%), and 21.2% of patients had light chain disease. Of the patients, 210 patients (17.8%) had serum creatinine level ≥ 2.0 mg/dL at diagnosis. Of the patients, 137 patients (26.5%) were classified as ISS I, 34.6% as ISS II, and 38.9% as ISS III. By applying the R-ISS, 213 (19.3%), 696 (62.9%), and 197 (17.8%) patients were assigned as stage I, II, and III, respectively. Chromosome analysis or FISH results were assessed in 1040 patients (88.4%), and 12.8% were classified as the high-risk cytogenetic group.

Overall, 424 patients (36.3%) received an IMid-based regimen as primary therapy, which is composed of thalidomide and dexamethasone (TD), or cyclophosphamide, thalidomide, and dexamethasone (CTD). Further, 371 patients (31.5%) received a proteasome inhibitor (PI)-based regimen as primary therapy, composed of bortezomib and dexamethasone (VD), or bortezomib, cyclophosphamide, and dexamethasone (VCD) or bortezomib, melphalan, and prednisone (VMP). Additionally, 103 patents (8.8%) received a combination regimen with PI and IMid as primary therapy, composed of bortezomib, thalidomide, and dexamethasone (VTD). Further, 261 patients (22.3%) received vincristine, doxorubicin, and dexamethasone (VAD) or cyclophosphamide and dexamethasone, or prednisolone as primary therapy. Four patients were treated with ixazomib, lenalidomide, and dexamethasone as primary therapy. One patient was treated with carfilzomib and one was treated with daratumumab.

During the entire treatment period, 903 patients (76.7%) underwent treatment with IMiDs such as thalidomide, lenalidomide or pomalidomide. Otherwise, 1010 patients (85.8%) were treated with PIs such as bortezomib, carfilzomib or ixazomib. Fifty-nine (5.0%) patients underwent daratumumab treatment. Autologous stem cell transplantation (ASCT) was performed in 495 patients (42.1%).

### Individual EASIX and survival outcomes

EASIX was calculated in all patients at diagnosis, and the median log2 EASIX score was 1.1 (IQR 0.3–2.3). The optimal EASIX cutoff value for OS was determined at 1.87 on the log2 scale using maximally selected log-rank statistics (95% CI 0.562–0.619, *p* < 0.001). Three hundred and seventy-two patients (31.6%) were classified as high EASIX (log2 EASIX > 1.87), and 805 (68.4%) were classified as low EASIX (log2 EASIX ≤1.87). Differences of the baseline clinical characteristics between the high EASIX group and low EASIX group patients are presented in Table 1. When compared with patients who had low EASIX, patients with high EASIX at diagnosis had a more advanced stage disease according to the ISS and R-ISS. High EASIX group patients had more adverse risk factors such as high-risk CA, poor performance score (PS), hypercalcemia, anemia, and renal insufficiency. Patients in the high EASIX group also received fewer ASCT than patients in the low EASIX group. There were no differences in the number of patients with cardiovascular disease or liver disease between the high EASIX group and low EASIX group (cardiovascular disease, 6.0% vs. 4.5%, *p* = 0.375; liver disease, 2.3% vs. 1.6%, *p* = 0.303).

**Table 1:** Comparison of baseline clinical characteristics (High EASIX vs. Low EASIX)

	High EASIX (log2 EASIX > 1.87) (*n* = 372)	Low EASIX (log2 EASIX ≤1.87) (*n* = 805)	*p*-value
Age > 65	170 (45.7)	359 (44.7)	0.753
Sex			< 0.001
Male	234 (62.9)	405 (50.3)	
Female	138 (37.1)	400 (49.7)	
Immunoglobulin (Ig) type			< 0.001
Ig G	162 (45.8)	453 (59.3)	
Ig M	2 (0.6)	5 (0.6)	
Ig A	67 (18.9)	168 (22.0)	
Ig D	11 (3.1)	13 (1.7)	
Light chain only	112 (31.6)	125 (16.4)	
ECOG PS ≥ 2	92 (24.7)	157 (19.5)	0.046
Calcium ≥10.2 mg/dl	103 (27.8)	82 (10.2)	< 0.001
Hb < 10.0 g/dl	273 (73.4)	389 (48.3)	< 0.001
Chromosomal abnormality			< 0.001
High risk	63 (16.9)	71 (8.8)	
Standard risk	262 (70.4)	650 (80.7)	
Non-assessable	47 (12.6)	84 (10.4)	
ISS			< 0.001
I	21 (5.6)	286 (35.5)	
II	73 (19.6)	328 (40.7)	
III	271 (72.8)	180 (22.4)	
Non-assessable	7 (1.9)	11 (1.4)	
R-ISS			< 0.001
I	7 (1.9)	206 (25.6)	
II	181 (48.7)	515 (64.0)	
III	162 (43.5)	35 (4.3)	
Non-assessable	22 (5.9)	49 (6.1)	
Year of diagnosis			0.073
2003–2005	24 (6.5)	79 (9.8)	
2006–2008	48 (12.9)	135 (16.8)	
2009–2011	84 (22.6)	168 (20.9)	
2012–2014	128 (34.4)	233 (28.9)	
2015–2017	88 (23.7)	190 (23.6)	
ASCT	128 (34.4)	367 (45.6)	< 0.001
Treatment regimen during entire treatment			
Thalidomide-based therapy	195 (52.4)	513 (63.7)	< 0.001
Lenalidomide-based therapy	153 (41.1)	461 (57.3)	0.612
Pomalidomide-based therapy	49 (13.2)	90 (11.2)	0.332
Bortezomib-based therapy	317 (85.2)	682 (84.7)	0.861
Carfilzomib-based therapy	35 (9.4)	77 (9.6)	1.000
Daratumumab-based therapy	19 (5.1)	40 (5.0)	1.000

*Abbreviations*: *n* Number, *ECOG PS* Eastern Cooperative Oncology Group Performance Status, *LDH* Lactate Dehydrogenase, *UNL* Upper limit of the normal value, *Hb* Hemoglobin, *ISS* International Staging System, *R-ISS* Revised-International Staging System, *ASCT* Autologous stem cell transplantation

**Fig. 1:**
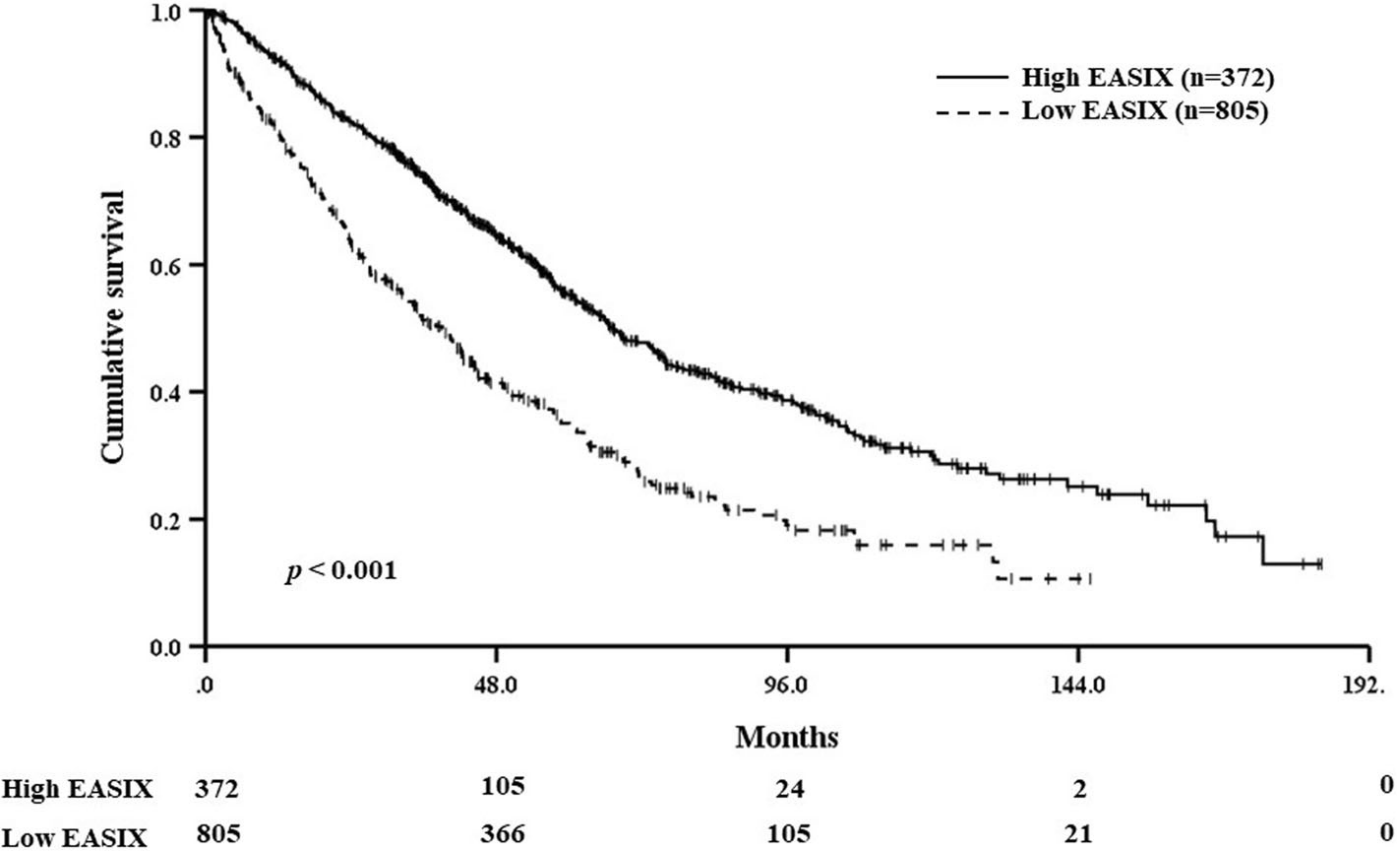
Kaplan-Meier survival curves for overall survival according to Endothelial Activation and Stress Index (EASIX) score

**Fig. 2:**
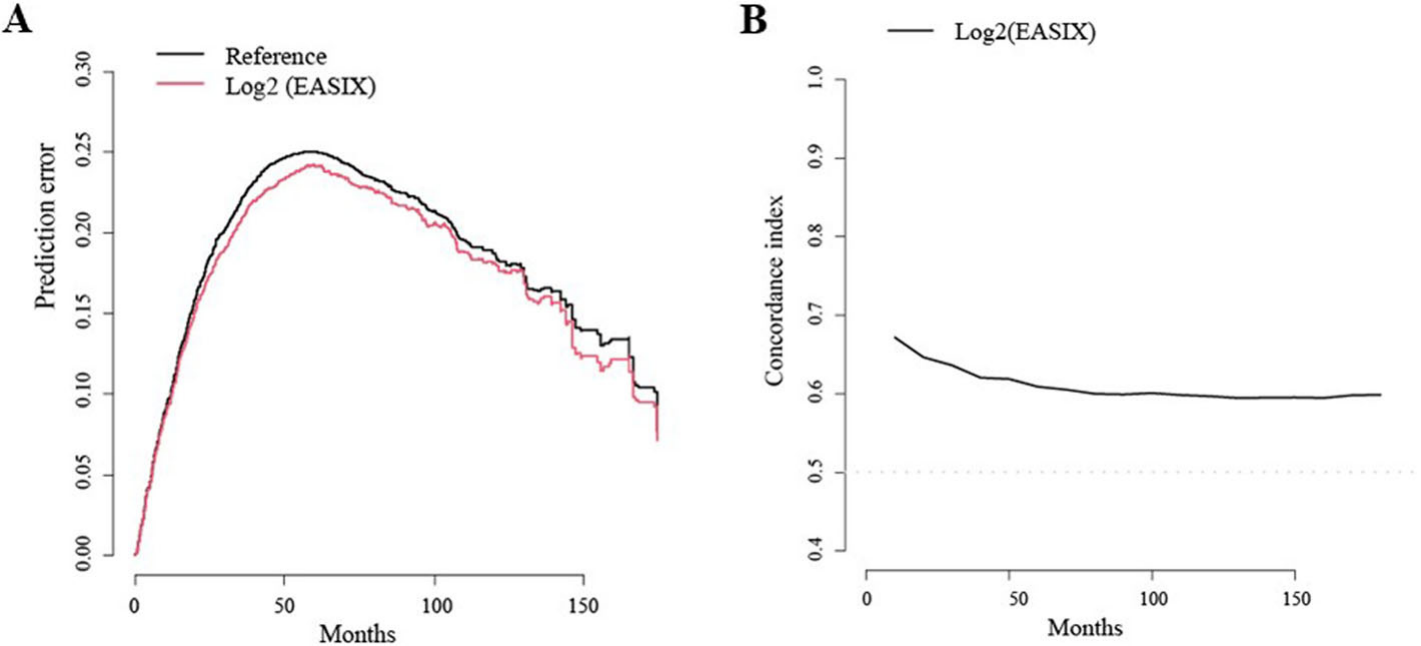
Prediction error curve (**a**) and time-dependent concordance index (**b**) for overall survival. A concordance index of 0.5 (dotted line) implies random concordance

After median follow-up for 50.0 months (range, 0.3–184.1), median OS was 58.2 months (95% CI 53.644–62.674). Patients with high EASIX score at diagnosis had significantly inferior OS compared to the patients with low EASIX [39.1 months (95% CI 34.1–44.1) vs. 67.2 months (95% CI 61.2–73.1), *p* < 0.001, Fig. 1]. We validated the prognostic value of EASIX for overall survival by calculating the prediction error curve and concordance index curve (Fig. 2). In the univariate Cox analysis, the risk of death was increased for high EASIX versus low EASIX (HR 1.878, 95% CI 1.600–2.205, *p* < 0.001). In multivariable analysis, including age, sex, ECOG PS, hemoglobin, calcium, EASIX, ISS, and high-risk CA, the risk of death was increased for patients aged more than 65 years (HR 1.476, 95% CI 1.245–1.750, *p* < 0.001), PS score greater than 1 (HR 1.495, 95% CI 1.240–1.802, *p* < 0.001), high EASIX (HR 1.444, 95% CI 1.170–1.780, *p* = 0.001), and high-risk CA (HR 1.565, 95% CI 1.241–1.973, *p* < 0.001). The univariate and multivariable Cox analysis results are summarized in Table 2. The univariate and multivariate Cox analysis including Log2 EASIX as a continuous variable showed that the Log2 EASIX could also predict survival outcome as a continuous variable (HR 1.189, 95% CI 1.113–1.269, *p* < 0.001, Supplementary Table 1).

**Table 2:** Univariate and multivariate Cox analysis for overall survival (*n* = 1177)

	Univariate			Multivariate		
	HR	95% CI	*p* - value	HR	95% CI	*p* - value
Age > 65	1.482	1.266–1.734	< 0.001	1.476	1.245–1.750	< 0.001
Sex (male)	1.178	1.008–1.377	0.039	1.097	0.926–1.299	0.284
ECOG PS ≥ 2	1.648	1.385–1.961	< 0.001	1.495	1.240–1.802	< 0.001
Hb < 10.0 g/dL	1.271	1.086–1.488	0.003	1.030	0.852–1.244	0.763
Calcium ≥10.2 mg/dL	1.372	1.117–1.686	0.003	1.124	0.896–1.409	0.312
Diagnosis at 2009–2014^a^	0.638	0.386–1.054	0.079			
Diagnosis at 2015–2017^a^	0.641	0.388–1.062	0.084			
Log2 EASIX > 1.87	1.878	1.600–2.205	< 0.001	1.444	1.170–1.780	0.001
ISS 2^b^	1.361	1.527–2.300	< 0.001	1.226	0.966–1.557	0.094
ISS 3^b^	1.874	1.101–1.682	< 0.001	1.309	1.000–1.714	0.050
High-risk CA	1.838	1.469–2.300	< 0.001	1.565	1.241–1.973	< 0.001

*Abbreviations*: *ECOG PS* Eastern Cooperative Oncology Group Performance Status, *Hb* Hemoglobin, *EASIX* Endothelial Activation and Stress Index, *ISS* International Staging System, *R-ISS* Revised-International Staging, System, *CA* Chromosomal abnormality

^a^ Diagnosis at 2003–2008 is the reference

^b^ISS 1 is the reference

**Fig. 3:**
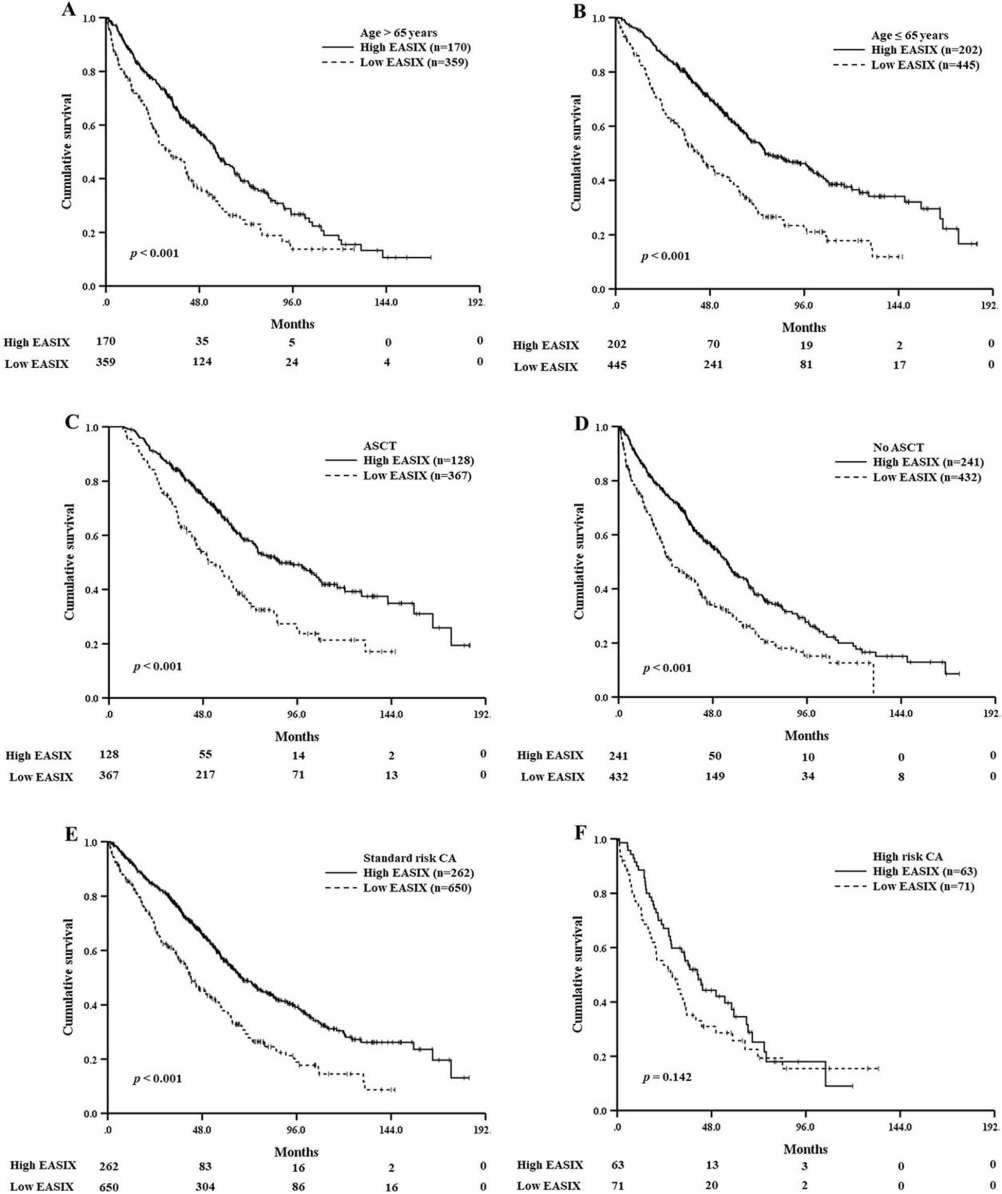
Kaplan-Meier survival curves for overall survival according to the Endothelial Activation and Stress Index (EASIX) score (**a**) in patients aged > 65 years, (**b**) in patients aged ≤65 years, (**c**) in patients who underwent autologous stem cell transplantation (ASCT), (**d**) in patients who did not receive ASCT, (**e**) in patients with standard cytogenetic abnormalities (CA), and (F) in patients with high-risk CA

Subgroup analyses for OS were also performed to define the prognostic role of EASIX in patients younger and older than 65 years of age, in patients who did and who did not receive ASCT, and in patients with high- and standard-risk CA. Patients with high EASIX showed significantly shorter OS than patients with low EASIX, regardless of age [Age > 65 years, 33.2 months (95% CI 23.8–42.7) vs. 56.5 months (95% CI 49.5–63.6), *p* < 0.001; Age ≤ 65 years, 42.1 months (95% CI 32.8–51.4) vs. 76.0 months (95% CI 60.4–91.6), *p* < 0.001, Fig. 3a and b], regardless of ASCT [ASCT, 52.8 months (95% CI 41.4–64.2) vs. 87.0 months (95% CI 69.5–104.6), *p* < 0.001; No ASCT, 26.9 months (95% CI 20.2–33.6) vs. 55.2 months (95% CI 48.4–62.0), *p* < 0.001, Fig. 3c and d]. Regarding CA, high EASIX was associated with poor OS in the standard-risk CA group [42.3 months (95% CI 35.8–48.9) vs. 68.4 months (95% CI 60.6–76.1), *p* < 0.001, Fig. 3e], but was not statistically significant in the high-risk CA group [28.1 months (95% CI 15.2–40.9) vs. 41.3 months (95% CI 31.4–51.2), *p* = 0.142, Fig. 3f].

### Prognostic impact of EASIX in each stage of ISS or R-ISS

**Fig. 4:**
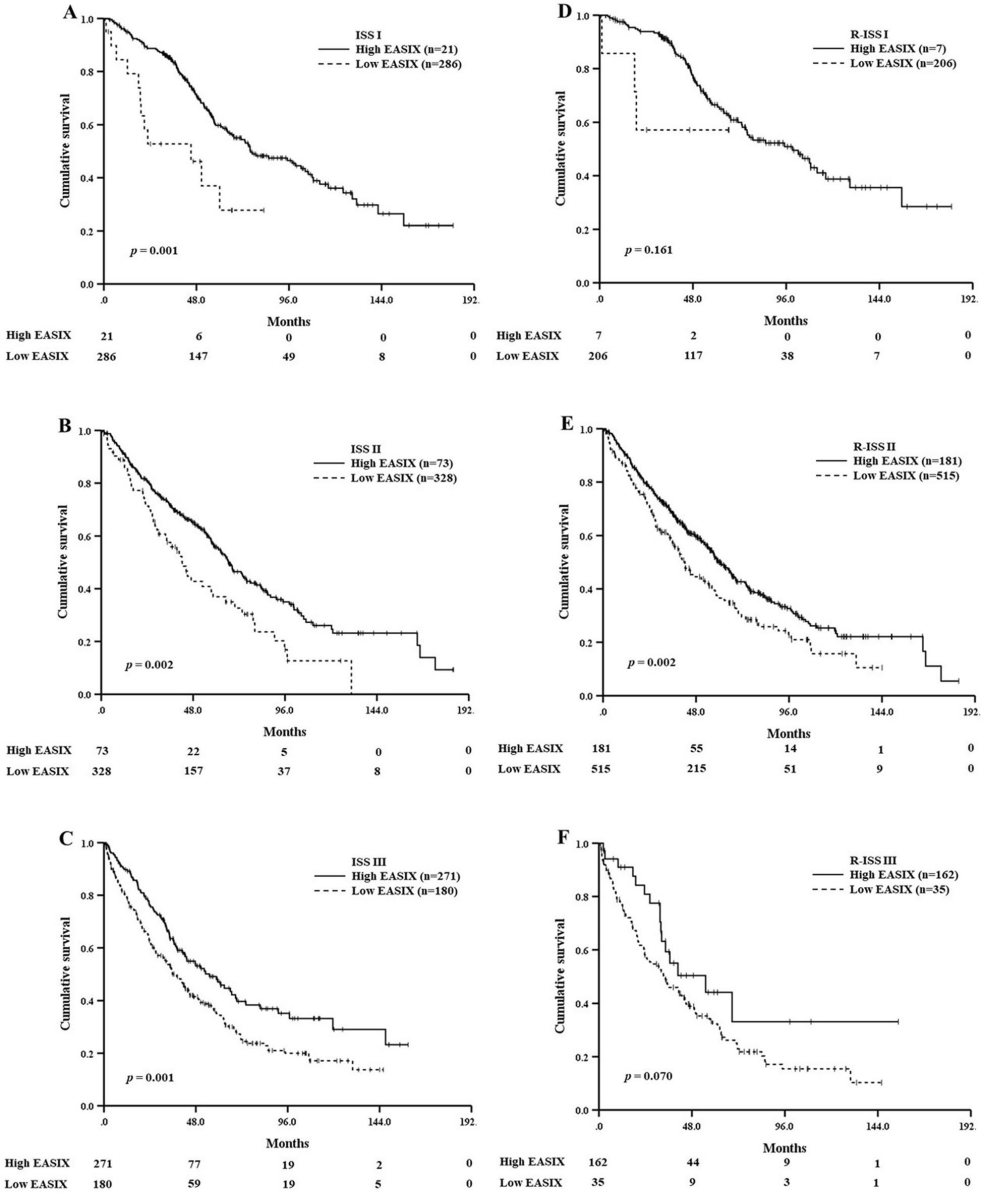
Kaplan-Meier survival curves for overall survival according to the Endothelial Activation and Stress Index (EASIX) score (**a**, **b**, **c**) in each subgroup of the International Staging System (ISS), and (**d**, **e**, **f**) in each subgroup of the Revised International Staging System (R-ISS)

This study analyzed whether EASIX could further stratify prognosis in more detail when integrated with ISS or R-ISS. In 307 patients with ISS I, 21 patients (6.8%) with high EASIX showed significantly inferior OS compared to other patients with low EASIX [45.2 months (95% CI 12.8–77.5) vs. 76.0 months (95% CI 54.7–97.3), *p* = 0.001, Fig. 4a]. In 401 patients with ISS II, 73 patients (18.2%) with high EASIX also showed significantly inferior OS compared to patients with low EASIX [42.3 months (95% CI 32.7–51.9) vs. 66.5 months (95% CI 58.8–74.2), *p* = 0.002, Fig. 4b]. In 451 patients with ISS III, 271 patients (60.1%) with high EASIX had significantly inferior OS than patients with low EASIX [36.8 months (95% CI 30.7–43.0) vs. 55.1 months (95% CI 40.2–70.0), *p* = 0.001, Fig. 4c]. Regarding R-ISS, OS was significantly different according to the EASIX group in R-ISS II [42.1 months (95% CI 35.5–48.8) vs. 61.0 months (95% CI 55.2–66.7), *p* = 0.002], but was not different in R-ISS I or R-ISS III [R-ISS I, not reached vs. 99.3 months (95% CI 72.3–126.2), *p* = 0.161; R-ISS III, 33.4 months (95% CI 22.3–44.5) vs. 55.1 months (95% CI 20.7–89.5), *p* = 0.070] (Fig. 4d, e, f).

## Discussion

This study showed that EASIX is a simple and powerful predictor of survival outcome in patients with newly diagnosed MM. Although fewer patients with high EASIX received ASCT than those with low EASIX, EASIX showed a prognostic value independent of ASCT. EASIX is a simple formula that can be calculated using platelet counts, serum creatinine, and LDH. These three variables as EASIX have been reported as a prognostic factor in MM. Elevated levels of serum LDH are associated with advanced disease and inferior survival outcomes in patients with MM who were treated with effective new agents such as thalidomide, lenalidomide, or bortezomib [11, 12]. Renal insufficiency at diagnosis is also associated with advanced disease stage and high tumor burden in MM [13, 14]. In addition, patients with renal insufficiency at diagnosis showed high risk of treatment-related toxicity and early mortality [15, 16]. Although development of new, effective agents improved the renal function and reduced early mortality [17, 18], a recent registry study showed that patients with renal insufficiency still had inferior survival outcomes compared to those with normal renal function [13]. The prognostic impact of platelet counts in MM is unclear. Platelet production, regardless of the degree of bone marrow plasmacytic infiltration, is probably affected by cytokines such as megakaryocyte growth factors, which are related to MM pathogenesis [19]. Further, MM patients who present with a low platelet count at diagnosis tend to have adverse prognosis [20–22]. As described in Table 1, the patients with high EASIX have more adverse clinical characteristics like hypercalcemia, anemia, poor performance status than the patients with low EASIX. And the patients with high EASIX have a significantly higher proportion of ISS III and R-ISS III than the patients with low EASIX. These mean that EASIX score reflects tumor burden and aggressiveness. Therefore, we considered that EASIX comprising these three variables could be useful to predict survival in MM.

EASIX was originally developed as an endothelial damage-related biomarker in patients with acute graft-versus-host disease (GVHD) after allogeneic stem cell transplantation. Luft et al. [5] first evaluated the prognostic role of EASIX in patients with acute GVHD after allogeneic stem cell transplantation, and demonstrated that patients with high EASIX showed a significantly higher non-relapsed mortality and inferior OS compared to those with low EASIX. A recent study showed that EASIX was associated with serological endothelial stress markers, especially angiopoieitin-2, and was significantly associated with poor OS in transplant-ineligible patients with low risk myelodysplastic syndrome [6]. This study suggested that EASIX could be a broadly applicable tool to predict prognosis independently of allogeneic stem cell transplantation. In MM, endothelial dysfunction and angiogenesis are important for disease progression and have prognostic potential. Endothelial cells in MM differently express cell adhesion molecules, receptors for cytokines, and growth factors compared to resting endothelial cells and these contribute to angiogenesis, which is essential for tumor growth, invasion, and metastasis [23–25]. Angiopeietin-2, an angiogenesis marker, is increased in MM and is associated with advanced disease and inferior survival [26, 27]. Therefore, EASIX may be important for the prognostic stratification of MM as an endothelial dysfunction-related marker independent of other prognostic factors.

This study showed that EASIX is useful to predict the survival in each group of ISS. ISS is a simple risk stratification system based on serum β2-microglobulin and albumin [28]; however, there was a concern for the prognostic value of ISS with respect to the introduction of new effective agents to treat MM. R-ISS was a new prognostic stratification system proposed by the International Myeloma Working Group [4]. The R-ISS stratifies patients into homogeneous survival subgroups by classifying patients with stage I and a poor prognosis, and patients with stage III and a better prognosis into stage II. Therefore, patients with R-ISS stage I and III had more homogenous survival outcomes, whereas patients with stage II were markedly increased and had heterogeneous survival outcomes [29, 30]. In this study, no significant difference in survival was observed according to EASIX in R-ISS I or III, but patients with high EASIX scores had significantly inferior survival than those with low EASIX. Thus, EASIX may be useful to further discriminate survival outcomes in each stage of ISS, or R-ISS II.

This study has some limitations. First, we do not have any data regarding progression-free survival (PFS). The clinical significance of PFS is growing in MM as many effective salvage treatment regimens including novel drugs are developed and affect OS prolongation. Further analysis about the association between EASIX and PFS could strengthen the prognostic role of EASIX. Second, we only evaluated EASIX score at the time of initial diagnosis. Assessment of EASIX at ASCT, disease progression, or recurrence might also be useful to analyze the prognostic value of EASIX. Third, there might be some limitations in the assessment of EASIX because platelet counts, creatinine, and LDH levels could be affected by several other conditions like heart problems, liver disease, or infection. Although the frequency of cardiovascular and liver disease was similar between the high EASIX and low EASIX group, we did not accurately analyze the effect of underlying disease on EASIX. Finally, this study cohort is heterogeneous because the diagnosis year is widely distributed and patients received variable induction and salvage treatment. Also, there is a possibility of over-fitting because of the lack of validation cohorts, and therefore the prognostic role of EASIX needs to be validated in further researches.

## Conclusions

This study firstly evaluated the prognostic impact of EASIX in patients with newly diagnosed MM. Patients with high EASIX at diagnosis had unfavorable characteristics, advanced disease stages, and showed significantly inferior survival outcomes compared to those with low EASIX. In addition, EASIX was useful to predict survival in each group of ISS or R-ISS II. Therefore, EASIX is a simple and powerful predictor of survival outcome in patients with newly diagnosed MM.

## Supplementary information

**Additional file 1: Supplementary Table 1.** Univariate and multivariate Cox analysis for overall survival (*n* = 1,177).

## Data Availability

The dataset used and analyzed during this study is available from the corresponding author on reasonable request.

## Abbreviations

ASCT: Autologous stem cell transplantation
CA: Chromosomal abnormalities
EASIX: Endothelial Activation and Stress Index
GVHD: Graft-versus-host disease
IMids: Immunomodulatory drugs
LDH: Lactate dehydrogenase
MGUS: Monoclonal gammopathy of undetermined significance
MM: Multiple myeloma
OS: Overall survival
PS: Performance score
PFS: Progression-free survival
R-ISS: Revised International Staging System
