# Supplementary material for: Endothelial activation and stress index (EASIX) is a reliable predictor for overall survival in patients with multiple myeloma
Source: BMC Cancer. 2020 Aug 24;20:803. doi: 10.1186/s12885-020-07317-y (PMC7446202; doi:10.1186/s12885-020-07317-y)
Supplement: Supplementary file 1 — Additional file 1: Supplementary Table 1. Univariate and multivariate Cox analysis for overall survival (n = 1,177). [file 12885_2020_7317_MOESM1_ESM.docx]

**Supplementary information**

**Endothelial Activation and Stress Index (EASIX) is a Reliable Predictor for Overall Survival in Patients with Multiple Myeloma**

Ga-Young Song^1^, Sung-Hoon Jung^1^, Kihyun Kim^2^, Seok Jin Kim^2^, Sang Eun Yoon^2^, Ho Sup Lee^3^, Mihee Kim^1^, Seo-Yeon Ahn^1^, Jae-Sook Ahn^1^, Deok-Hwan Yang^1^, Hyeoung-Joon Kim^1^, and Je-Jung Lee^1^

^1^Department of Hematology-Oncology, Chonnam National University Hwasun Hospital, Hwasun, Jeollanamdo, Republic of Korea; ^2^Samsung Medical Center, Seoul, Republic of Korea; ^3^Kosin University Gospel Hospital, Busan, Republic of Korea

**Running title:** EASIX in multiple myeloma

**Corresponding author:**

**Sung-Hoon Jung, M.D., Ph.D.,** Department of Hematology-Oncology, Chonnam National University Hwasun Hospital, 322 Seoyangro, Hwasun, Jeollanamdo 519-763, Republic of Korea; Tel: 82-61-379-7636 ; Fax: 82-61-379-7628 ; E-mail: [shglory@hanmail.net](mailto:shglory@hanmail.net)

**Kihyun Kim, M.D., Ph.D.,**

Division of Hematology-Oncology, Department of Medicine, Samsung Medical Center, Sungkyunkwan University School of Medicine, 81 Irwon-ro, Gangnam-gu, Seoul, Republic of Korea; Tel +82-2-3410-3456, Fax:+82-2-3410-1757, Email: kihyunkimk@gamil.com

**Supplementary table 1. Univariate and multivariate Cox analysis for overall survival (n = 1,177)**

|  | Univariate | | | Multivariate | | |
| --- | --- | --- | --- | --- | --- | --- |
|  | HR | 95% CI | *p* - value | HR | 95% CI | *p* - value |
| Age > 65 | 1.482 | 1.266-1.734 | <0.001 | 1.467 | 1.238-1.739 | <0.001 |
| Sex (male) | 1.178 | 1.008-1.377 | 0.039 | 1.071 | 0.904-1.269 | 0.426 |
| ECOG PS ≥ 2 | 1.648 | 1.385-1.961 | <0.001 | 1.477 | 1.224-1.781 | <0.001 |
| Hb < 10.0 g/dL | 1.271 | 1.086-1.488 | 0.003 | 1.016 | 0.840-1.230 | 0.868 |
| Calcium ≥ 10.2 mg/dL | 1.372 | 1.117-1.686 | 0.003 | 1.143 | 0.913-1.431 | 0.242 |
| Diagnosis at 2009-2014^#^ | 0.638 | 0.386-1.054 | 0.079 |  |  |  |
| Diagnosis at 2015-2017^#^ | 0.641 | 0.388-1.062 | 0.084 |  |  |  |
| Log2 EASIX^§^ | 1.255 | 1.194-1.318 | <0.001 | 1.189 | 1.113-1.269 | <0.001 |
| ISS 2* | 1.361 | 1.527-2.300 | <0.001 | 1.210 | 0.953-1.536 | 0.118 |
| ISS 3* | 1.874 | 1.101-1.682 | <0.001 | 1.169 | 0.890-1.536 | 0.262 |
| High-risk CA | 1.838 | 1.469-2.300 | <0.001 | 1.581 | 1.255-1.993 | <0.001 |

Abbreviations: ECOG PS, Eastern Cooperative Oncology Group Performance Status; Hb, Hemoglobin; EASIX,

Endothelial Activation and Stress Index; ISS, International Staging System; R-ISS, Revised-International Staging

System; CA, Chromosomal abnormality

^#^ Diagnosis at 2003-2008 is the reference

^§^ Log2 EASIX as a continuous variable

*ISS 1 is the reference
